# Fish consumption and cognitive function in aging: a systematic review of observational studies

**DOI:** 10.1007/s11357-026-02188-w

**Published:** 2026-03-15

**Authors:** Justyna Godos, Giuseppe Caruso, Agnieszka Micek, Alberto Dolci, Carmen Lili Rodríguez Velasco, Evelyn Frias-Toral, Jason Di Giorgio, Nicola Veronese, Andrea Lehoczki, Mario Siervo, Zoltan Ungvari, Giuseppe Grosso

**Affiliations:** 1 Department of Biomedical and Biotechnological Sciences, University of Catania, 95123 Catania, Italy; 2 Departmental Faculty of Medicine, UniCamillus-Saint Camillus International University of Health and Medical Sciences, Rome, Italy; 3 IRCCS San Camillo Hospital, Venice, Italy; 4 Statistical Laboratory, Faculty of Health Sciences, Jagiellonian University Medical College, 31-501, Cracow, Poland; 5 Sustainable Development Department, Bolton Food SpA, 20124 Milan, Italy; 6 Research Group on Food, Nutritional Biochemistry and Health, Universidad Europea del Atlántico, Isabel Torres 21, 39011 Santander, Spain; 7 Universidad Internacional Iberoamericana, Arecibo, PR 00613 USA; 8 Faculty of Health Science, Universidade Internacional do Cuanza, Cuito, Bié, Angola; 9 Escuela de Medicina, Universidad Espíritu Santo, Samborondón, 0901952 Ecuador; 10 Division of Research, Texas State University, 601 University Dr, San Marcos, TX 78666 USA; 11 Saint Camillus International, University of Health Sciences, Rome, Italy; 12 International Training Program in Geroscience, Doctoral College/Institute of Preventive Medicine and Public Health, Semmelweis University, Budapest, Hungary; 13 Curtin-Chulalongkorn Collaborative Centre for Nutrition and Food Research and Education, Curtin University, Perth, WA Australia; 14 Faculty of Health Sciences, School of Population Health, Curtin University, Perth, WA 6102 Australia; 15 Curtin Dementia Centre of Excellence, Enable Institute, Curtin University, Perth, WA 6102 Australia; 16 Curtin Medical Research Institute (CMRI), Curtin University, Perth, WA 6102 Australia; 17 Vascular Cognitive Impairment, Neurodegeneration and Healthy Brain Aging Program, Department of Neurosurgery, University of Oklahoma Health Sciences Center, Oklahoma City, OK USA

**Keywords:** Fish, Seafood, Cognitive function, Memory, Aging

## Abstract

Epidemiological studies consistently link higher fish intake with slower rates of cognitive decline and lower dementia incidence. The aim of the present study was to systematically review existing observational studies investigating the association between fish consumption and cognitive function in older adults. A total of 25 studies (8 cross-sectional and 17 prospective including mainly healthy older adults, age range of participants ranging from 18 to 30 years at baseline in prospective studies to 65 to 91 years, representing the upper limit of the age spectrum) were reviewed. Cognitive functions currently investigated in most published studies included various domains, such as global cognition, memory (episodic, working), executive function (planning, inhibition, flexibility), attention and processing speed. Existing studies greatly vary in terms of design (cross-sectional and prospective), geographical area, number of participants involved, and tools used to assess the outcomes of interest. The main findings across studies are not univocal, with some studies reporting stronger evidence of association between fish consumption and various cognitive domains, while others addressed rather null findings. The most consistently responsive domains were processing speed, executive functioning, semantic memory, and global cognitive ability among individuals consuming fish at least weekly, which are highly relevant to both neurodegenerative and vascular forms of cognitive impairment. Positive associations were also observed for verbal memory and general memory, though these were less uniform and often attenuated after multivariable adjustment. In contrast, associations with reaction time, verbal-numerical reasoning, and broad composite scores were inconsistent, and several fully adjusted models showed null results. In conclusion, the evidence suggests that regular fish intake (typically ≥1–2 servings per week) is linked to preserved cognitive performance, although some inconsistent findings require further investigations.

**Graphical Abstract:**

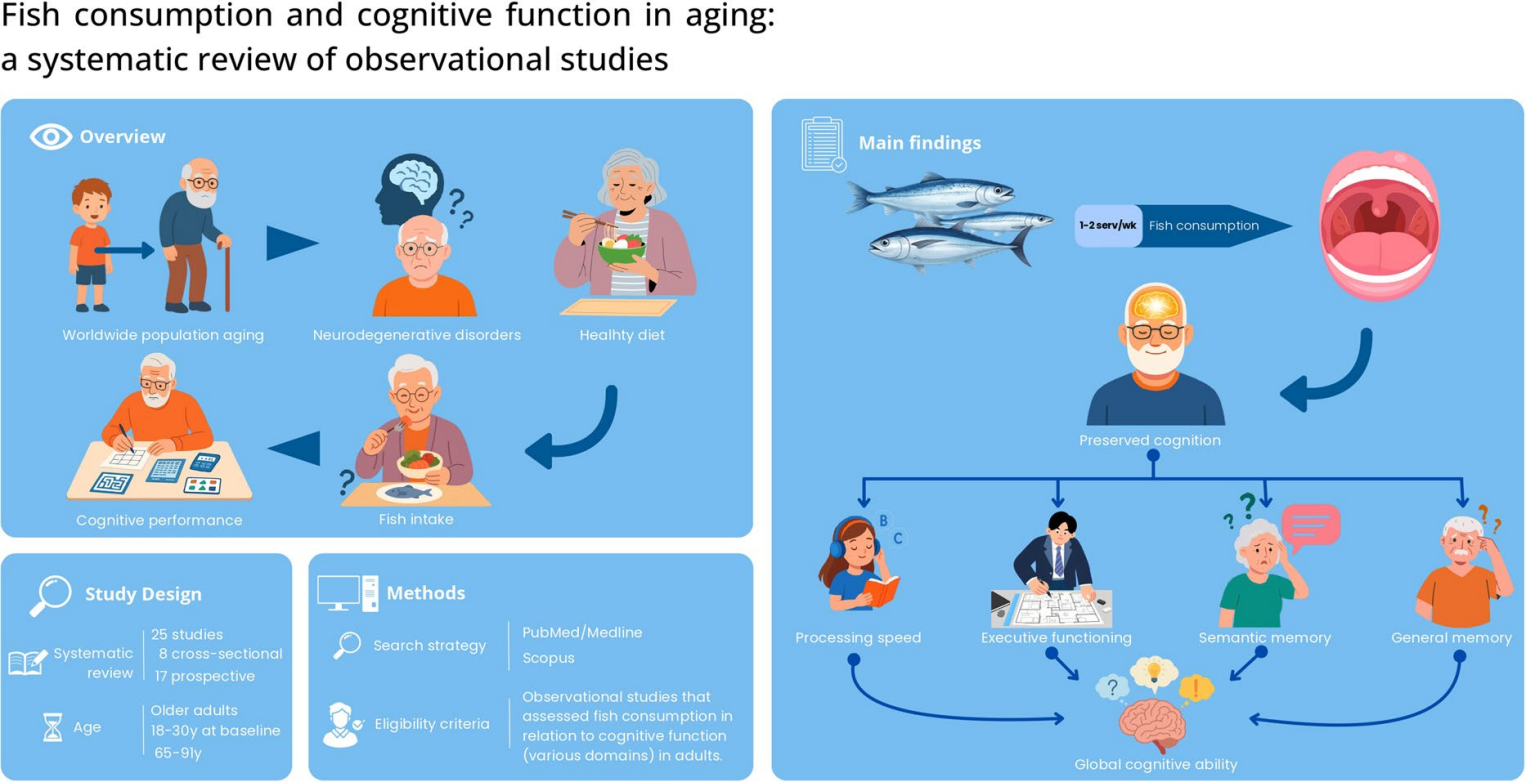

**Supplementary Information:**

The online version contains supplementary material available at 10.1007/s11357-026-02188-w.

## Introduction

The rapid aging of the global population is fundamentally transforming the epidemiology of late-life cognitive disorders [1]. The Global Burden of Disease (GBD) 2019 analysis estimated 57.4 million people living with dementia in 2019 and projected this number to increase to approximately 153 million by 2050, despite relatively stable age-standardized prevalence rates [2]. Current projections suggest that with the growing old population worldwide, the global prevalence of people with dementia is deemed to increase from roughly 50 million in 2019 to about 150 million by 2050 [2]. Dementia is already among the leading causes of death globally and accounts for nearly 10 million new cases annually, with Alzheimer’s disease contributing to over 50% of cases [3]. Importantly, population-based and neuropathological studies show that over half of dementia cases also involve a substantial vascular component, suggesting multiple etiological targets to address in order to reduce the future burden of disease [4].

Lifestyle factors are now understood as major modifiable determinants of late-life cognitive trajectories, with diet quality being considered an important driver of cognitive health in the aging population [5]. Prospective cohorts and meta-analyses consistently associate higher adherence to healthy dietary patterns with lower risk of cognitive decline and dementia [6]. Dietary models characterized by plant-based foods (i.e., rich in vegetables, fruits, legumes, whole grains) as well as healthy sources of animal proteins (including white meat and fish), including the Mediterranean diet, the MIND diet (a Mediterranean-DASH hybrid emphasizing leafy greens, berries, nuts, olive oil, and fish while limiting red/processed meats and sweets), and the DASH pattern (designed for blood-pressure control) have been associated with lower risk of dementia [7–9], multidomain trials that include dietary guidance alongside exercise, vascular risk management, and cognitive training have produced significant benefits on global cognition, supporting diet as a key component of effective prevention packages [11]. In line with this literature, the World Health Organization’s guideline on risk reduction recommends healthy dietary patterns, together with physical activity, smoking cessation, and vascular risk control, as part of an integrated strategy to delay cognitive decline and dementia [12].

Various components of healthy diets may explain their role in preserving cognitive function and maintaining brain health [13]. A common feature across healthy dietary patterns associated with reduced dementia risk is the regular inclusion of seafood, particularly fish [14]. Fish consumption has been associated with various health benefits, primarily related to cardiovascular health and certain cancers, among others [15, 16]. Fish is a source of high-quality protein and long-chain omega-3 polyunsaturated fatty acids (PUFAs), which also play essential roles in brain structure and function, influencing membrane fluidity, synaptic plasticity, and neuroinflammation [17, 18]. Summary evidence from epidemiological studies consistently links higher fish consumption with slower rates of cognitive decline and lower dementia incidence [19]. Beyond omega-3 PUFAs, seafood provides additional neuroprotective nutrients such as vitamin D, selenium, iodine, and B vitamins, which collectively support neuronal integrity, neurotransmitter synthesis, and vascular health [20, 21], suggesting that whole-food consumption of seafood within a broader dietary pattern may confer benefits beyond isolated supplementation [22, 23].

While a substantial body of evidence links fish consumption with overall cognitive health and reduced dementia risk, no previous review has comprehensively summarized findings across specific cognitive domains in relation to fish intake. This represents a critical gap, as cognitive decline and dementia syndromes often manifest heterogeneously across domains, and dietary components may differentially influence these functions. The aim of the present study was to systematically review and synthesize published human studies investigating the association between fish consumption and cognitive performance in older adults.

## Methods

The guidelines for biomedical research of a consensus framework for design, conduct, and reporting were followed [24]. The conceptualization and reporting of this study followed the Meta-analyses Of Observational Studies in Epidemiology (MOOSE) guidelines (Table S1) [25]. The systematic review protocol was registered in the PROSPERO International Prospective Register of Systematic Reviews database (ID: CRD42024501232, at https://www.crd.york.ac.uk/prospero/).

### Search strategy

A comprehensive literature search was performed across two major electronic databases (PubMed/Medline and Scopus) from inception to March 2024, using combinations of keywords and MeSH terms related to fish, seafood, and shellfish and cognitive outcomes (Table S2). Eligibility criteria for the systematic review and meta-analysis were specified using the PICOS criteria (Table S3). In particular, eligible studies included observational studies (cross-sectional, case-control, retrospective, and prospective) that assessed fish consumption in relation to cognitive function in adults. Studies were included in the systematic review if they considered cognitive function as the main outcome, including various domains such as global cognition, memory (episodic, working), executive function (planning, inhibition, flexibility), attention, and processing speed. Only English language studies were eligible. Reference lists of all eligible studies were also examined for any additional studies not previously identified. Two authors (J.G. and A.M.) independently screened titles and abstracts, assessed full texts against eligibility criteria, and extracted data, with discrepancies resolved by consensus.

### Data extraction and quality assessment

Qualitative and quantitative data from all included studies were collected using a standardized electronic form. The following information was extracted: first author name and publication year, study name and design, study location, sample size, population age and sex, details on the assessment method of dietary habits and the exposure, details on the assessment method of the outcome of interest and the outcome of interest, main findings of the study with measures of relation.

The methodological quality of each eligible study was independently assessed by two authors (J.G. and A.M.) using the Newcastle-Ottawa Scale (NOS) [26], any incongruity was resolved through a discussion and reaching consensus. The NOS scoring system comprises 3 domains of quality (selection, comparability, and outcome) and allows us to assess study characteristics depending on the type of study design. In particular, studies scoring over 5 and 7 points for cross-sectional and prospective studies, respectively, were deemed to be of good/high quality.

## Results

### Study selection

The systematic search identified 1169 potentially eligible studies, out of which 1039 were selected for title/abstract and full text examination (Fig. S1). A total of 813 studies were excluded based on title/abstract evaluation and 201 for not meeting at least one of the eligibility criteria, leaving a final selection of 25 studies [27–51] to be included and fully reviewed.

### Study characteristics

The included studies varied in terms of design (9 cross-sectional [27–35] and 16 prospective [36–51]), geographical area (13 studies conducted in Europe [27–32, 36–42], 9 in the US [33, 43–49], 3 in Asian countries [34, 50, 51], and 1 in Australia [35]), number of participants involved (with the majority including relatively large samples ranging between 189 and 30,484 participants), age ranges (ranging from 18 to 30 years at baseline in prospective studies to 65 to 91 years, representing the upper limit of the age spectrum) summarized by domain in Table 1. The included studies used various tools to assess the outcomes of interest and in particular various cognitive domains (Table S4). The quality assessment (Table S5, Table S6) revealed stronger evidence among a few cross-sectional studies [27, 28, 33] and higher-quality prospective cohort studies [37, 43, 48], which consistently achieved Newcastle-Ottawa Scale scores in the moderate-to-high range (typically 6–8 points), reflecting strong cohort representativeness, appropriate exposure ascertainment, adequate follow-up duration, and rigorous outcome assessment. Most studies demonstrated strong comparability through adjustment for key confounders, further strengthening the validity of their findings. However, weaker evidence was observed in cross-sectional studies and prospective studies with lower quality scores (typically 4–5 points), which often lacked adequate control for confounding, had less robust exposure assessment, or insufficient follow-up duration [30–32, 35].

**Table 1:** The main characteristics of the studies included in the systematic review (n = 25)

Author, year	Cohort name (country)	Design, follow-up	Population sex, age	Dietary assessment	Exposure	Outcome assessment	Main findings
Sasaki, 2024 [33]	NHANES 2011–2014 (US)	Cross-sectional, NA	3123 MF, 60–80 y	24-h dietary recall, dietary interview	Fish consumption none 1 meal/mo 2 meals/mo 3–5 meals/mo ≥ 6 meals/mo	CERAD, DSST	Consumption of 2 or more fish meals per month was associated with significantly higher cognitive function scores for both CERAD and DSST cognitive tests, when compared with not consuming any fish. Also, when considering age and sex, there were significantly higher cognitive scores with increased fish consumption, for participants aged 66–74 y as well as for female participants
Zhang, 2021 [42]	UKWCS (UK)	Prospective, 10–15 y	503 F, 62 ± 6.6 y	217-item FFQ	Total fish consumption as continuous; roasted/baked, fried, barbecued/grilled fish non-consumers consumers	CRT, SRT	There was no significant difference in energy-adjusted total fish daily intake between fast and slow groups for both SRT and CRT. Higher fish consumption was not significantly linearly associated with the odds of fast reaction time. Consumers of roasted/baked fish were 46% more likely to be in the slow SRT group (adjusted OR 1.46, 95% CI 1.00, 2.13) when compared to non-consumers
Mao, 2019 [47]	CARDIA (US)	Prospective, over 25 y	3231 MF, 50.1 ± 3.6 y, 18–30 y at baseline	Diet history questionnaire	Quintiles of non-fried seafood consumption: Ql ≤ 0.29 serv/d Q2 0.30–0.55 serv/d Q3 0.56–0.88 serv/d Q4 0.89–1.43 serv/d Q5 > 1.43 serv/d	RAVLT, DSST, Stroop Test	Compared to the lowest non-fried seafood consumption, the highest non-fried seafood consumption was associated with better results in DSST (MD = 2.87; 95% CI: 1.29, 4.45; *p*_*trend*_ < 0.01) and the Stroop test (MD = −1.72; 95% CI: −2.81, −0.62; *p*_*trend*_ < 0.01), yet in the most adjusted model the significant findings remained only for DSST (MD = 1.48; 95% CI: −0.05, 3.01; *p*_*trend*_ = 0.04). Non-fried seafood consumption was not associated with RAVLT scores
Zhu, 2018 [50]	SMHS, SWHS (China)	Prospective, 14.4 y (average)	30,484 MF, 70–88 y (at the functional status assessment)	FFQ	Fish consumption median: Q1 10 g/d for M, 8 g/d for F Q2 22 g/d for M, 20 g/d for F Q3 35 g/d for M, 32 g/d for F Q4 54 g/d for M, 51 g/d for F Q5 97 g/d for M, 94 g/d for F	Self-reported questionnaire on impairments in walking capability, hearing/vision, memory, and decision-making ability	Compared to the lowest quintile of fish consumption, those with the highest fish consumption were less likely to have impaired walking capability (OR = 0.79, 95% CI: 0.69, 0.89), serious memory decline (OR = 0.73, 95% CI: 0.62, 0.86), and having always difficulty in making decisions (OR = 0.75, 95% CI: 0.65, 0.87)
Samieri, 2018 [43]	3 C (France); NHS, WHS, CHAP, MAP (US)	Prospective, 3.9–9.1 y (median)	23,688 MF, ≥ 65 y	FFQ	Fish consumption: < 1 serv/wk 1 serv/wk 2–3 serv/wk ≥ 4 serv/wk	3 C study: MMSE, Isaacs’ Set Test, BVRT; CHAP: MMSE, SDMT, CERAD, WMS-R, EBMT; MAP: MMSE, CFT, BNT, NART, DST-B, DST-F, DOT; NHS: TICS, CFT, DST-B, EBMT; WHS: TICS, CFT, EBMT	When considering pooled cohort results a significant trend for increasing intake of fish was associated with slower global cognitive decline, but not for individual cohorts. Similarly to global cognition, the results showed a significant trend of increasing fish intake associated with decreasing rate of episodic memory decline for pooled analysis. Also, a statistically significant lower rate of memory decline for individuals consuming ≥ 4 servings of fish per week vs < 1 serving per week was observed (MD = 0.018, 95% CI: 0.004, 0.032 standard units)
Nooyens, 2018 [36]	DCS (The Netherlands)	Prospective, 5y	2612 MF, 55.3 ± 6.9 y, 43–70 y	178-item SQ-FFQ	- Total fish intake < 1/mo 1/mo—1/wk 1—2/wk ≥ 2/wk - Fatty fish intake 0 0–1/mo 1/mo—1/wk ≥ 1/wk	15 Words VLT, the Stroop Colour- Word Test, the Word Fluency test, and the Letter Digit Substitution Test	No association between total fish or fatty fish consumption and change in global cognitive function, speed or flexibility over time was observed. However, individuals with low fatty fish consumption (less than once a week) showed slower memory decline than those with no fatty fish consumption
Brouwer-Brolsma, 2018 [29]	NQplus (The Netherlands)	Cross-sectional, NA	1607 MF, 52.9 ± 12 y, 20–70 y	183-item SQ-FFQ	Fish and seafood intake as continuous variable	LFT, SDMT, SRT	After adjustment for age, sex and educational level, fish and seafood consumption was positively linearly associated with the LFT score (unstandardised *β* = 0.012 points, *p* =.02 per 1 g/d), however the association attenuated after additional adjustment for demographic, lifestyle, and nutritional factors. Processing speed, and everyday memory was not associated with fish and seafood consumption
Del C Valdés Hernández, 2017 [38]	LBC1936 (UK)	Prospective, 3 y	189 MF, 72.7 ± 0.8 y (whole sample—866 persons from wave 2)	175-item SQ-FFQ	Intake of different types of fish (white fish, oily fish, shellfish, fish products, fish canned) as continuous variable	Six subtests of the WAIS-IIIUK (Digit Symbol, Digit Span Backward, Symbol Search, Letter-Number Sequencing, Block Design & Matrix Reasoning)	In the subgroup of people representing opposite ends of the iodine intake spectrum there was the positive linear association between general memory and the canned fish consumption (*β* = 0.2, *p* = 0.003)
van de Rest, 2016 [48]	MAP (US)	Prospective, 4.9 ± 2.5 y (mean)	915 MF, 81.4 ± 7.2 y	144-item SQ-FFQ	Seafood < 1 meal/wk ≥ 1 meal/wk	MMSE, Logical Memory, East Boston Story, WLM, WLR, WLRc, BNT, VFT, 15-item reading test, DST‐F, DST‐B, Digit Ordering, Symbol Digit Modalities Test, Number Comparison, Stroop Neuropsychological Screening Test, JLO, SPM	Consumption of seafood was associated with slower decline in semantic memory and perceptual speed (*β* = 0.024, *p* = 0.03 and *β* = 0.020, *p* = 0.05 for consumers at least 1 vs < 1 meal per week). In APOE ε4 carriers, weekly consumers of seafood demonstrated slower rates of decline in global cognition and in multiple cognitive domains (episodic memory, semantic memory, and perceptual speed) compared with consumers of seafood less than once a week
Crichton, 2015 [46]	MSLS (US)	Prospective, 18 ± 5.3 y (mean)	333 MF, 60.5 ± 12.8 y	37-item FFQ	Fish consumption as continuous variable	WAIS (Verbal—Information, Vocabulary, Comprehension, Arithmetic, Digit Span, Similarities; Performance—Picture Completion, Object Assembly, Block Design, Picture Arrangement, Digit Symbol Substitution)	After adjustment for age, gender, education, income, ethnicity, CES-D, smoking, physical activity, BMI, CRP, folic acid, SBP, and DBP, fish consumption was linearly positively associated with total WAIS score and performance score (unstandardised *β* = 0.011, SE = 0.003 and *β* = 0.018, SE = 0.005, respectively, both *p* < 0.01)
Qin, 2014 [51]	CHNS (China)	Prospective, 5.3 y (mean)	1566 MF, 63 ± 6 y	3-day 24-h dietary recall	- Total fish consumption (fresh and preserved fish and shellfish): < 1 serv/wk ≥ 1 serv/wk - Fresh fish consumption: < 1 serv/wk ≥ 1 serv/wk	Telephone Interview for Cognitive Status–modified	Among participants aged at least 65 years, after adjustment for demographic and socioeconomic covariates, multiple lifestyle and dietary components, the rate of cognitive decline was consistently slower for those who consumed at least 1 serving/wk of fish in total compared with < 1 serving/wk fish (*β* = 0.37 point, 95% CI: 0.11, 0.56; *p* = 0.003). After restriction only to fresh fish the results were similar
Kesse-Guyot, 2014 [40]	SU.VI.MAX 2 (France)	Prospective, 13.0 y (mean)	2430 MF, 65.6 ± 4.5 y	Bimonthly 24-h dietary records, self-administrated questionnaire	Fish and seafood consumption: < 2 times/wk ≥ 2 times/wk	DST‐B, DST‐F, TMT, RI-48 cued recall test, VFT	Infrequent fish consumption (less than 2 times per week) was inversely associated with global cognitive performance (MD = −0.79, *p* = 0.05) and executive functioning (MD = −0.86, * p* = 0.03), although after adjusting for other covariates the associations did not remain significant (MD = −0.61, *p* = 0.12 and MD = −0.66, *p* = 0.11, respectively)
Danthiir, 2014 [35]	EPOCH (Australia)	Cross-sectional, NA	390 MF, 73.1 ± 5.5 y, 65–91 y	38-item SQ-FFQ	- Fish, oily fish, white fish current consumption: never < 1 times/mo 1–3 times/mo 1 time/wk 2 times/wk 3–4 times/wk 1 time/day ≥ 2 times/day - Past consumption for childhood, early adulthood, adulthood, and middle-age life periods: daily 2–3 times/wk 2–3 times/mo rarely or never	KFRCT^#^, SPM +, EPT, Retrieval Fluency, Face Memory, Word Memory, DSST, Counting Span, Operation Span, Inspection Time, SRT, CRT, OMO, SMS, RAVLT, Simon Task, Colour ST, Spatial ST, FT, Simple MT, Up MT, Diagonal MT	In cognitively normal older adults no evidence of a beneficial effect of increased fish intake on cognitive performance was found, but higher frequency of current total fish consumption was associated with slower cognitive speed reflected in simple/choice reaction time and inhibition tests (after adjustment for age, sex, education, apoE-ε4 carrier status, smoking, language, current and early-life income level, alcohol intake, energy intake, vigorous activity level, medication). Worse inhibitory processes and reasoning speed were linked with greater oily fish consumption and psychomotor speed and simple/choice reaction time was negatively predicted by frequency of consumption of white fish. A small negative effect of fish intake in childhood on cognitive function was also observed
Samieri, 2013 [44]	NHS (US)	Prospective, 6 y	16,058 F, 74.3 ± 2.3 y	116-item SQ-FFQ	Fish consumption (in quintiles of intake, servings not specified)	TICS, EBMT, CFT, DST‐B	Greater intake of fish was significantly associated with higher multivariable-adjusted mean verbal memory score in later life (*p*_*trend*_ = 0.0003, MD = 0.04, 95% CI: 0.01, 0.08 for Q5 vs Q1)
van de Rest, 2009 [49]	NAS (US)	Prospective, 6 y	1025 M, 68 y	126-item SQ-FFQ	Quartiles of fatty fish intake, median (range) Q1 0.21 serv/wk (−0.02–0.38) Q2 0.80 serv/wk (0.66–0.90) Q3 1.25 serv/wk (1.11–1.38) Q4 2.79 serv/wk (1.76–3.61)	VFT, BNT short and CP (CERAD); Pattern comparison and CPT (NES2) DST‐B and Vocabulary (WAIS-R)	No significant associations between fish consumption and cognition were observed neither in cross-sectional (baseline cognition) nor longitudinal analysis (6-y cognitive change)
Dangour, 2009 [31]	OPAL (UK)	Cross-sectional, NA	867 MF, 74.7 ± 2.6 y, 70–79 y	Individual questions	Fish consumption treated as a continuous variable	CVLT, SRT, CRT, story recall, VFT, letter cancellation, location memory, DSST, DST-B, DST-F	Increasing trend of the CVLT mean scores by higher fish consumption was revealed in unadjusted analysis, however this association did not remain significant after adjustment for age, gender and reported age at leaving full-time education and GHQ-30 score. Similarly, fish consumption was positively related to the scores of global cognition, memory, executive function and delay only in unadjusted analysis, but not after multivariable adjustment
Nurk, 2007 [27]	HUSK, Norway	Cross-sectional, NA	2031 MF, 70–74 y	169-item SQ-FFQ	Total fish, fatty fish, lean fish, processed fish, fish sandwich consumption: < 10 g/d ≥ 10 g/d	KOLT, TMT-A, m-DST, m-BD, m-MMSE, COWAT S-task	Individuals with at least 10 g/d total intake of fish had higher mean test scores and a lower prevalence of poor cognitive performance compared to those whose intake was less than 10 g/d. The dose-dependent associations between total intake of seafood and cognition were detected with the maximum effect at an intake of 75 g/d. Fish intake positively affected most cognitive functions. The strongest effect was observed for unprocessed lean fish and fatty fish
Kalmijn, 2004 [28]	DCS, The Netherlands	Cross-sectional, NA	1613 MF, 56.3 ± 7.1 y	178-item SQ-FFQ	Total fish and fatty fish consumption (1-SD increase corresponding to 4.0 g/d)	VLT, CST, SCWT, LDST, CFT	Consumption of fatty fish was significantly inversely associated with risk of impairment of overall cognition and cognitive speed (OR, 95% CI: 0.77, 0.60–0.97 and 0.71, 0.55–0.92 per 1 SD increase, respectively). There were no associations between fatty fish and memory and cognitive flexibility, as well as adjusted mean change in daily intake of total fish did not differ between normal and cognitive impaired persons
Fieldhouse, 2020 [30]	NUDAD, The Netherlands	Cross-sectional, NA	357 MF, 65 ± 8.3 y	34-item SQ-FFQ	Fish consumption as 0–10 scale being a component of dietary quality score	VAT (CFT, naming condition), RAVLT, SCWT, TMT-A, VOSP, DST‐F, DST‐B, FAB, LFT	No significant correlation was found between fish consumption and global cognition, and its individual domains, including memory, language, visuospatial functioning, attention or executive functioning
Rauchmann, 2023 [32]	DELCODE, Germany	Cross-sectional, NA	938 MF, 70.8 ± 5.9 y, 59–88 y	148-item SQ-FFQ	Fish consumption: daily consumption or multiple times per week once per week, less than once per week or never	Composite memory factor score (MEM score)	No significant correlation between fish consumption and MEM score, indicative of cognitive functioning, was found (*β* = 0.05, *p* = 0.17 for multiple times per week or more frequent vs at most once per week fish consumption)
Kesse-Guyot, 2011 [39]	SU.VI.MAX 2, France	Prospective, 7.5 y	3294 MF, 64.2 ± 4.7 y	Bimonthly 24-h dietary records	Fish and seafood consumption (in quartiles of intake, servings not specified)	MMSE, CDS, 5-word test	A marginally significant (*p* = 0.1) trend of lower risk of cognitive difficulties across higher fish consumption categories was found with OR = 0.80, 95% CI: 0.63–1.01 for Q4 versus Q1 group. Fish consumption was not associated with poor scores on the global cognitive test and verbal memory test
Kim, 2013 [45]	WHS, US	Prospective, 5.6 y (mean)	5988 F, 71.8 ± 4.0 y	FFQ	- Total seafood: < 1.0 serv/wk 1.0 serv/wk 1.1–2.0 serv/wk > 2.0 serv/wk - Tuna and other dark-meat fish: < 1.0 serv/wk 1.0 serv/wk > 1.0 serv/wk - Light-meat fish: < 1.0 serv/wk 1.0 serv/wk > 1.0 serv/wk - Shellfish: < 1.0 serving/wk ≥ 1.0 serving/wk	TICS, EBMT, CFT (CERAD)	Having one serving of tuna and other dark-meat fish per week was significantly associated with lower odds of worst verbal memory change, when compared to consuming less than once-weekly (OR = 0.76, 95% CI: 0.60, 0.96; *p* = 0.02). However, different amounts of total seafood consumption were not significantly associated with either changes in global cognition or verbal memory
Fischer, 2018 [41]	AgeCoDe, Germany	Prospective, 10 y	2622 MF, 81.2 ± 3.4 y	8-item questionnaire	Fresh fish consumption: never < 1 time/wk 1 time/wk several times/wk every day	CERAD (Word List Immediate Recall, Word List Delayed Recall, Word List Recognition)	Longitudinal joint modelling of incident AD and memory decline revealed no correlation between higher fresh fish consumption and both outcomes (*β* = −0.03, 95% CI: −0.14, 0.08, *p* = 0.610 for memory decline)
Ylilauri, 2022 [37]	KIHD, Finland	Prospective, 4 y	482 M, 54–60 y	Food recording of 4 days	Quartiles of fish intake, median (range): Q1 0 g/d (< 3) Q2 18 g/d (3–31) Q3 48 g/d (32–66) Q4 102 g/d (> 66)	MMSE, TMT-A,VFT, the Selective Reminding Test, and the Russell’s adaptation of the Visual Reproduction Test	Fish consumption wasn’t linked to performance in the MMSE, TMT-A and Visual Reproduction Test. Positive association was observed between intake of fish and a score of the Selective Reminding Test (*p*_*trend*_ = 0.04). Subjects in the highest fish intake tertile scored 1.9 points more compared to those in the lowest tertile. In APOE-ε4 carriers higher fish intake was directly associated with a better performance in the VRT (5.8 more words on average produced by individuals in T3 compared with T1 group), but there was no such relationship in the non-carriers
Huang, 2023 [128]	CHNS, China	Prospective, 3 y (median)	4066 MF, 62.2 ± 7.0 y	3-day 24 h dietary recall	Tertiles of fish and shellfish intake, mean (range) T1 6.1 g/d (< 100) T2 128.0 g/d (100–189) T3 275.8 g/d (≥ 190)	TICS-m	Multivariate linear analysis showed that higher combined fish and shellfish consumption was associated with better global cognitive function when compared to low consumption (*β* = 0.104, 95% CI: 0.048, 0.160 for high vs low consumption, and *β* = 0.083, 95% CI: 0.033, 0.132 for moderate vs low consumption, *p*_*trend*_ = 0.002)

*3C*, Three-City; *AgeCoDe*, German Study on Aging, Cognition and Dementia in Primary Care Patients; *AGES*-*RS*, Age Gene/Environment Susceptibility-Reykjavik Study; *BD*, Block Design; *BNT*, Boston Naming Test; *BVRT*, Benton Visual Retention Test; CARDIA, Coronary Artery Risk Development in Young Adults; *CCSNSD*, Community-based Cohort Study on Nervous System Diseases; *CDS*, McNair’s Cognitive Difficulties Scale; *CERAD*, Consortium to Establish a Registry for Alzheimer’s Disease; *CFT*, Category Fluency Test; CHAP, Chicago Health and Aging Project; *CHNS*, China Health and Nutrition Survey; *COWAT*, Controlled Oral Word Association Test; *CP*, Constructional Praxis; *CPT*, Continuous Performance Test; *CRT*, Choice Reaction Time; *CST*, Concept Shifting Test; *DCS*, Doetinchem Cohort Study; *DELCODE*, DZNE-Longitudinal Cognitive Impairment and Dementia Study; *DOT*, Digit Ordering Test; *DSST*, Digit Symbol Substitution Test; *DST*, Digit Symbol Test; *DST-B*, Digit Span Backward Test; *DST-F*, Digit Span Forward Test; *E3N*, Etude Epidémiologique de Femmes de la Mutuelle Générale de l’Education Nationale; *EBMT*, East Boston Memory Test; *EPOCH*, Older People, Omega-3, and Cognitive Health; *EPT*, Everyday Problems Test; F, female; *FAB*, Frontal Assessment Battery; *FFQ*, Food Frequency Questionnaire; *FT*, Flanker Task; HELIAD, Hellenic Longitudinal Investigation of Ageing and Diet; *HUSK*, Hordaland Health Study; *JLO*, Judgment of Line Orientation; *KFRCT*, Kit of Factor-Referenced Cognitive Tests; *KIHD*, Kuopio Ischaemic Heart Disease Risk Factor Study; *KOLT*, Kendrick Object Learning Test; *LBC1936*, Lothian Birth Cohort 1936; *LDST*, Letter Digit Substitution Test; *LFT*, Letter Fluency Test; *M*, male; *m-*, modified; *MAP*, Rush Memory and Aging Project; *MMSE*, Mini-Mental State Examination; *mo*, month; *MoCA*, Montreal Cognitive Assessment; *MSLS*, Maine Syracuse Longitudinal Study; *MT*, Movement Time; *NA*, not applicable; *NART*, National Adult Reading Test; *NAS*, Normative Aging Study; *NES2*, Neurobehavioral Evaluation System; *NHS*, Nurses’ Health Study; *NQplus*, Nutrition Questionnaires plus; *NR*, not reported; *NUDAD*, Nutrition the Unrecognized Determinant for Alzheimer’s Disease; *OMO*, Odd-Man-Out; *OPAL*, Older People And n-3 Long-chain polyunsaturated fatty acid; *RAVLT*, Rey Auditory Verbal Learning Test; *SCWT*, Stroop Color and Word Test; *SDMT*, Symbol Digit Modalities Test; *SMHS*, Shanghai Men’s Health Study; *SMS*, Sternberg Memory Scanning; *SPM*, Standard Progressive Matrices; *SPM + (*Standard Progressive Matrices Plus; *SQ-FFQ*, Semi-Quantitative Food Frequency Questionnaire; *SRT*, Simple Reaction Time; *ST*, Stroop Task; *SU.VI.MAX*, Supplémentation en Vitamines et Minéraux Antioxydants; *SWHS*, Shanghai Women’s Health Study; *TICS*, Telephone Interview for Cognitive Status; *TMT-A*, Trail Making Test, part A; *TMT-B*, Trail Making Test, part B; *UKWCS*, UK Women’s Cohort Study; *VFT*, Verbal Fluency Test; *VLT*, Verbal Learning Test; *VOSP*, Visual Object and Space Perception; *WAIS*, Wechsler Adult Intelligence Scale; *WAIS-R*, Wechsler Adult Intelligence Scale Revised; *WHS*, Women’s Health Study; *WLM*, Word List Memory; *WLR*, Word List Recall; WLRc, Word List Recognition; *WMS-R*, Wechsler Memory Scale-Revised; *ZES*, Zutphen Elderly Study

^#^*KFRCT*: Letter Sets, First and Last Names, Finding As, Number Comparison and Word Endings

### Summary findings from cross-sectional studies

Among investigations conducted in the European region including larger samples, a cross-sectional study from the Hordaland Health Study (HUSK), conducted in Western Norway, investigated the relationship between seafood consumption and cognitive performance in 2,031 adults aged 70–74 years. Dietary intake was assessed using a modified version of a comprehensive 169-item semi-quantitative FFQ able to distinguish between fish sandwiches, fatty fish, lean fish, and processed fish. Cognitive outcomes were evaluated using a test battery including the Kendrick Object Learning Test (KOLT), Trail Making Test, part A (TMT-A), modified version of the Digit Symbol Test (m-DST), modified version of the Block Design (m-BD), modified version of the Mini-Mental State Examination (m-MMSE), and abridged version (S-task) of the Controlled Oral Word Association Test (COWAT). Results showed that participants with a total fish intake of at least 10 g/day had higher mean test scores and a lower prevalence of poor cognitive performance compared to those with lower intakes. Regarding specific domains, all but m-MMSE scores were higher in individuals with higher intake of both fatty and lean fish. Furthermore, dose-dependent associations were observed between total seafood intake and cognitive function, with the maximum effect at an intake of 75 g/day, with the strongest association detected for unprocessed lean fish and fatty fish [27]. A cross-sectional study conducted within the Doetinchem Cohort Study (DCS) in the Netherlands examining dietary intakes of 1,613 men and women (mean age 56.3 ± 7.1 years) using a validated 178-item semi-quantitative FFQ, and cognitive outcomes measured with a test battery including the Verbal Learning Test (VLT), Concept Shifting Test (CST), Stroop Color-Word Test (SCWT), Letter-Digit Substitution Test (LDST), and Category Fluency Test (CFT), showed that fatty fish consumption was significantly and inversely associated with the risk of impaired overall cognition and psychomotor speed, although no associations were observed between fatty fish intake and memory or cognitive flexibility [28]. Furthermore, a cross-sectional study including 1607 adults aged 20–70 years from the central part of Netherlands assessing habitual dietary intake with a 183-item food frequency questionnaire and cognitive performance with the Letter Fluency Test (LFT), Symbol Digit Modalities Test (SDMT) and Story Recall Test (SRT) showed a positive linear association between fish and seafood consumption and the Letter Fluency Test score in the less adjusted models, then attenuated after adjustment for demographic, lifestyle, and nutritional factors, while no further associations with processing speed or everyday memory were observed [29]. In contrast, another cross-sectional study using baseline data from the Nutrition the Unrecognized Determinant for Alzheimer’s Disease (NUDAD) study in the Netherlands including 357 men and women (mean age 65 ± 8.3 years) using a 34-item semi-quantitative food frequency questionnaire to assess fish consumption and cognitive performance using a set of tests, including the Visual Association Test (VAT), including the Category Fluency Test (CFT) and naming condition, the Rey Auditory Verbal Learning Test (RAVLT), the Stroop Color and Word Test (SCWT), Trail Making Test part A (TMT-A), the Visual Object and Space Perception battery (VOSP), Digit Span Forward (DST-F) and Digit Span Backward (DST-B) tests, the Frontal Assessment Battery (FAB), and the Letter Fluency Test (LFT) showed that fish consumption was not significantly associated with global cognitive performance or any of its individual domains, including memory, language, visuospatial functioning, attention, or executive function [30]. Also, a cross-sectional analysis using baseline data from the Older People And n-3 Long-chain Polyunsaturated Fatty Acid (OPAL) study, another randomized controlled trial conducted in the United Kingdom investigating the relationship between fish consumption and cognitive function in 867 participants (men and women) aged 70–79 years (mean 74.7 ± 2.6 years), in which dietary fish intake was assessed through individual questions and categorized into five groups, from once or less a month to more than once a week, distinguishing between white and oily fish, and cognitive outcomes were measured using Californian Verbal Learning Test (CVLT), Simple Reaction Time (SRT), Choice Reaction Time (CRT), story recall, Verbal Fluency Test (VFT), letter cancellation, location memory, Digit Symbol Substitution Test (DSST), Digit Span Backward Test (DST-B), and Digit Span Forward Test (DST-F), showed in unadjusted analyses an increasing trend in CVLT scores and positive associations with global cognition, memory, executive function, and delayed recall across higher fish consumption categories. However, these associations were no longer significant after adjustment for age, sex, age at leaving full-time education, and GHQ-30 score [31]. Finally, a cross-sectional analysis from the German Center for Neurodegenerative Disorders (Deutsches Zentrum für Neurodegenerative Erkrankungen, DZNE)-Longitudinal Cognitive Impairment and Dementia Study (DELCODE) including 938 participants explored the relation between dietary factors, memory performance and hippocampal atrophy in healthy older adults and individuals at risk for Alzheimer’s disease. Dietary intake was assessed using a semi-quantitative food frequency questionnaire (FFQ) with 148 food items, and cognitive functioning was evaluated through a comprehensive neuropsychological battery covering multiple domains summarized into a composite memory factor score (MEM) indicative of cognitive functioning, actually showing no relation with fish consumption [32].

Only one cross-sectional study has been conducted in the US. Data from the National Health and Nutrition Examination Survey (NHANES) including 3123 adults aged ≥ 60 years testing cognitive performance using the CERAD Word List Learning test for immediate and delayed recall and the Digit Symbol Substitution Test (DSST) for executive function showed that consumption of two or more fish meals per month was associated with significantly higher cognitive scores compared to no fish consumption [33].

Another study was conducted in Asian countries, an analysis of the Community-based Cohort Study on Nervous System Disease including 4309 Chinese adults aged ≥ 55 years assessing dietary intake with a validated semi-quantitative food frequency questionnaire and cognitive performance with the Montreal Cognitive Assessment (MoCA), and the memory index score (MIS), executive index score (EIS), visuospatial index score (VIS), language index score (LIS), attention index score (AIS), and orientation index score (OIS) were applied to evaluate the cognitive domain function of memory, execution, visuospatial, language, attention, and orientation, respectively, showed that subjects with higher fish consumption tended to have higher scores of global cognitive function and all domains investigated, and to have lower odds of mild cognitive impairment (MCI) [34]. Ultimately, the cross-sectional baseline data from the Older People, Omega-3, and Cognitive Health (EPOCH) study, a randomized controlled trial conducted in Australia, examined 390 men and women (mean age 73.1 ± 5.5 years, range 65–91) in which dietary fish intake was assessed using a 38-item semi-quantitative FFQ, with a mean weekly fish consumption of 2.0 ± 1.4 servings (including 0.8 ± 0.8 servings of oily fish and 1.2 ± 1.0 servings of white fish), and cognitive outcomes were evaluated using a comprehensive test battery including Kit of Factor-Referenced Cognitive Tests (KFRCT subtests: Letter Sets, First and Last Names, Finding As, Number Comparison, and Word Endings), Standard Progressive Matrices Plus (SPM +), Everyday Problems Test (EPT), Retrieval Fluency, Face Memory, Word Memory, Digit Symbol Substitution Test (DSST), Counting Span, Operation Span, Inspection Time, Simple Reaction Time (SRT), Choice Reaction Time (CRT), Odd-Man-Out (OMO), Sternberg Memory Scanning (SMS), Rey Auditory Verbal Learning Test (RAVLT), Simon Task, Colour ST, Spatial ST, Flanker task (FT), Simple Movement Time (Simple MT), Up Movement Time (Up MT), and Diagonal Movement Time (Diagonal MT). In older adults with normal cognitive function, higher frequency of total fish consumption was associated with slower cognitive speed, with greater consumption of oily fish linked to poorer inhibitory control and slower reasoning speed, while higher intake of white fish was associated with slower psychomotor speed and longer reaction times [35].

### Summary findings from prospective studies

Several studies have been conducted in European cohorts. A study including 2612 adults aged 43–70 years from the Doetinchem Cohort Study (DCS) assessing dietary intake through a 178-item FFQ and cognitive function at baseline and 5-year follow-up, testing memory, information processing speed and cognitive flexibility with the 15 Words Verbal Learning, Stroop Color-Word, Word Fluency and Letter Digit Substitution tests, showed no consistent associations between (fatty) fish intake and cognitive decline in a population with relatively low fish consumption, while indicating faster memory decline in persons consuming fatty fish less than once per week compared with those consuming it more frequently [36]. A prospective analysis from the Kuopio Ischaemic Heart Disease Risk Factor Study (KIHD) including 482 men with 4 year follow-up assessed diet by 4 day food records and cognition using the Mini-Mental State Examination (MMSE), Trail Making Test A (TMT-A), Verbal Fluency Test, Selective Reminding Test (SRT), and Russell’s adaptation of the Visual Reproduction Test (VRT). The results showed that fish consumption was not associated with MMSE, TMT-A, or VRT scores. Higher fish intake was positively associated with SRT performance. Among APOE-ε4 carriers, higher fish intake was linked to better VRT performance, with no association in non-carriers [37]. Another prospective study including 189 participants from the Lothian Birth Cohort 1936 investigating estimated dietary iodine intake, brain structural measurements and cognitive abilities, assessing diet with the 175-item Scottish Collaborative Group Food Frequency Questionnaire and deriving general cognitive ability from six WAIS-IIIUK subtests (Digit Symbol, Digit Span Backward, Symbol Search, Letter-Number Sequencing, Block Design, Matrix Reasoning), showed in individuals at opposite extremes of iodine intake a positive linear association between general memory performance and canned fish consumption [38]. A prospective study conducted within the Supplémentation en Vitamines et Minéraux Antioxydants (SU.VI.MAX 2) randomized controlled trial in France included 3,294 men and women (mean age 64.2 ± 4.7 years) who were followed for 7.5 years. Dietary intake was assessed using bimonthly 24-h dietary records, with fish and seafood consumption categorized into quartiles. Cognitive performance was evaluated with the Mini-Mental State Examination (MMSE), McNair’s Cognitive Difficulties Scale (CDS), and the 5-word test. Results showed a marginally significant trend toward a lower risk of cognitive difficulties across higher fish consumption categories (OR = 0.80, 95% CI: 0.63, 1.01 for Q4 versus Q1). However, fish consumption was not associated with poor performance on the global cognitive test or the verbal memory test [39]. However, another prospective analysis from the same cohort (SU.VI.MAX 2) including 2,430 men and women (mean age 65.6 ± 4.5 years), with a longer follow-up duration of 13.0 ± 0.7 years and other tools used to test cognitive performance, including the Digit Span Backward Test (DST-B), Digit Span Forward Test (DST-F), Trail Making Test (TMT), RI-48 cued recall test, and Verbal Fluency Test (VFT) reported that participants with a lower frequency of fish intake (< 2 times per week) showed poorer global cognitive performance (MD = −0.79, *p* = 0.05) and executive function (MD = −0.86, *p* = 0.03). However, these associations were attenuated and lost statistical significance after adjustment for covariates (MD = −0.61, *p* = 0.12 and MD = −0.66, *p* = 0.11 respectively) [40]. Besides, a prospective study conducted within the German Study on Aging, Cognition and Dementia in Primary Care Patients (AgeCoDe) included 2,622 men and women (mean age 81.2 ± 3.4 years), who were followed for 10 years. Dietary exposure was assessed using an 8-item questionnaire focusing on fresh fish consumption. Cognitive performance was evaluated with the Word List Immediate Recall, Word List Delayed Recall, and Word List Recognition, all derived from the Consortium to Establish a Registry for Alzheimer’s Disease (CERAD) neuropsychological battery. Results showed that, based on longitudinal joint modeling of incident Alzheimer’s disease and memory decline, higher fresh fish consumption was not significantly associated with either outcome [41]. Also prospective longitudinal analysis of the UK Women’s Cohort Study, involving 503 women with an average age of 62 years, assessing dietary intakes with a 217-item FFQ and testing the simple reaction time (SRT) and choice reaction time (CRT) showed no significant difference in energy-adjusted total fish daily intake between the fast and slow groups for SRT or CRT and demonstrated that roasted/baked fish consumers were 46% more likely to be in the slow SRT group and that higher fish consumption per 100 g was not significantly linearly associated with the odds of a faster reaction time [42].

Among studies conducted in the American population, a pooled analysis from an American prospective study of older adults from five cohorts including the Three-City (3C) Study, the Nurses’ Health Study (NHS), the Women’s Health Study (WHS), the Chicago Health and Aging Project (CHAP), and the Rush Memory and Aging Project (MAP) (accounting for a total of 23,688 participants) aimed to assess the relationship of fish intake to cognitive decline and examining interactions with genes related to Alzheimer’s disease. The study found that higher fish intake was associated with slower global and episodic memory decline, with ≥ 4 servings/week linked to lower rates of decline compared with < 1 serving/week [43]. Among other studies investigating in-depth the individual cohorts, a prospective analysis from the Nurses’ Health Study (NHS) examined 16,058 women (mean age 74.3 ± 2.3 years) to assess associations between long-term adherence to the Mediterranean diet and cognitive function. Dietary intake was measured using a 116-item semi-quantitative food frequency questionnaire (SQ-FFQ), with responses ranging from “never or < 1 time/month” to “ ≥ 6 times/day” and standardized portion sizes for all foods. Fish consumption was categorized into quintiles, with median intakes of 1.4 to 3.5 servings per week. Cognitive function was assessed via trained interviewers using validated telephone tests, including the Telephone Interview for Cognitive Status (TICS), the East Boston Memory Test (EBMT), the Category Fluency Test (CFT), and the Digit Span Backwards Test (DST-B). Results showed that greater fish intake was significantly associated with higher multivariable-adjusted mean verbal memory scores in later life (*p* for trend = 0.0003; MD = 0.04, 95% CI: 0.01, 0.08 for Q5 vs. Q1) [44]. Also, another prospective analysis conducted within the Women’s Health Study (WHS) included 5,988 women (mean age 71.8 ± 4.0 years) who were followed for an average of 5.6 years. Dietary intake was assessed using a food frequency questionnaire (FFQ), with fish and seafood consumption categorized as follows: total seafood (< 1.0 serving/week, 1.0 serving/week, 1.1–2.0 servings/week, > 2.0 servings/week); tuna and other dark-meat fish (< 1.0 serving/week, 1.0 serving/week, > 1.0 serving/week); light-meat fish (< 1.0 serving/week, 1.0 serving/week, > 1.0 serving/week); and shellfish (< 1.0 serving/week, ≥ 1.0 serving/week). Cognitive outcomes were evaluated with the Telephone Interview for Cognitive Status (TICS), East Boston Memory Test (EBMT), and the Category Fluency Test (CFT) from the Consortium to Establish a Registry for Alzheimer’s Disease (CERAD). Results showed that consuming one serving per week of tuna and other dark-meat fish was significantly associated with better verbal memory compared with consuming less than once weekly (OR = 0.76, 95% CI: 0.60, 0.96; *p* = 0.02). However, different levels of total seafood consumption were not significantly associated with changes in either global cognition or verbal memory [45]. Within the Maine Syracuse Longitudinal Study (MSLS), a prospective study investigated 333 men and women (mean age 60.5 ± 12.8 years) followed for an average of 18.0 ± 5.3 years. Dietary intake was assessed using a 37-item food frequency questionnaire (FFQ), with fish consumption considered as a continuous variable. Cognitive outcomes were evaluated using the Wechsler Adult Intelligence Scale (WAIS), including verbal subtests (Information, Vocabulary, Comprehension, Arithmetic, Digit Span, Similarities) and performance subtests (Picture Completion, Object Assembly, Block Design, Picture Arrangement, Digit Symbol Substitution). The results showed that, after adjustment for age, gender, education, income, ethnicity, CES-D, smoking, physical activity, body mass index (BMI), C-reactive protein (CRP), folic acid, systolic blood pressure (SBP), and diastolic blood pressure (DBP), fish consumption was linearly and positively associated with both total WAIS score (*β* = 0.011, SE = 0.003; *p* < 0.01) and performance score (*β* = 0.018, SE = 0.005; *p* < 0.01) [46]. The Coronary Artery Risk Development in Young Adults (CARDIA), a multicenter longitudinal cohort study including 3,231 US adults aged 18–30 years assessing cognitive function using the Rey Auditory Verbal Learning Test (RAVLT), the Digit Symbol Substitution Test (DSST), and the Stroop Test, while collecting dietary intake through the CARDIA Diet History questionnaire. The results showed that higher non-fried seafood consumption was associated with better DSST and Stroop performance, with associations for DSST remaining significant after multivariable adjustment [47]. A prospective cohort study using data from the Rush Memory and Aging Project (MAP) in the US, included 915 men and women (mean age 81.4 ± 7.2 years) with a mean follow-up of 4.9 ± 2.5 years. Intake of seafood was recorded using a validated 144-item semi-quantitative FFQ, with consumption grouped into < 1 or ≥ 1 meals per week. Cognitive outcomes were evaluated using a comprehensive battery including the Mini-Mental State Examination (MMSE), Logical Memory, East Boston Story, Word List Memory (WLM), Word List Recall (WLR), Word List Recognition (WLRc), Boston Naming Test (BNT), Verbal Fluency Test (VFT), 15-item reading test, Digit Span Forward Test (DST-F) Digit Span Backward Test (DST-B), Digit Ordering, Symbol Digit Modalities Test, Number Comparison, Stroop Neuropsychological Screening Test, Judgment of Line Orientation (JLO), and Standard Progressive Matrices (SPM). Results showed that seafood consumption of at least 1 meal/week was associated with slower decline in semantic memory (*β* = 0.024, *p* = 0.03) and perceptual speed (*β* = 0.020, *p* = 0.05) compared with consumption of less than 1 meal/week. Among APOE ε4 carriers, weekly seafood consumers demonstrated slower rates of decline in global cognition and in multiple cognitive domains, including episodic memory, semantic memory, and perceptual speed, compared with those consuming seafood less than once per week [48]. However, among other studies conducted in the US, one reported null findings: a prospective analysis from the Normative Aging Study (NAS) investigated the association between fatty fish intake and cognitive performance as well as 6-year cognitive change in 1,025 men with a mean age of 68 years. Dietary fish intake was assessed using a 126-item semi-quantitative food frequency questionnaire, from which fatty fish intake was specifically evaluated. Cognitive outcomes were measured using the Verbal Fluency Test (VFT), Boston Naming Test short version (BNT short) and Constructional Praxis (CP) from the Consortium to Establish a Registry for Alzheimer’s Disease (CERAD); Pattern Comparison and Continuous Performance Test (CPT) from the NES2 battery; and Digit Span Test-Backward (DST-B) and Vocabulary from the Wechsler Adult Intelligence Scale Revised (WAIS-R). Results showed no significant associations between fish consumption and various cognitive domains in longitudinal analyses of 6-year cognitive change [49].

Among cohorts conducted in Asian countries, a prospective study using data from two population-based cohorts, the Shanghai Women’s Health Study (1996–2015) and the Shanghai Men’s Health Study (2002–2015), including 30,484 adults followed for an average of 14.4 years and aged 70–86 years at functional assessment, using food-frequency questionnaires to assess habitual diet and four questions to evaluate impairments in walking, hearing/vision, memory, and decision-making, showed that higher fish consumption was associated with lower odds of impaired walking capability, serious memory decline, and difficulty in decision-making compared with the lowest intake [50]. Also, a prospective cohort study conducted within the China Health and Nutrition Survey (CHNS) in China included 1,566 men and women (mean age 63.0 ± 6.0 years), with a mean follow-up duration of 5.3 years. Dietary intake was assessed using 3-day 24-h dietary recalls, categorizing total fish consumption (fresh and preserved fish and shellfish) as < 1 serving/week or ≥ 1 serving/week, and fresh fish consumption as < 1 serving/week or ≥ 1 serving/week. Cognitive outcomes were evaluated using the Telephone Interview for Cognitive Status-modified. The results showed that, among participants aged at least 65 years, after adjustment for demographic and socioeconomic covariates as well as multiple lifestyle and dietary components, the rate of cognitive decline was consistently slower for those who consumed at least 1 serving/week of total fish compared with < 1 serving/week (*β* = 0.37 points, 95% CI: 0.11, 0.56; *p* = 0.003). Similar associations were observed when the analysis was restricted to fresh fish consumption only. Furthermore, in secondary analysis, the rate of verbal memory score decline among infrequent consumers was 2.5 times faster than those consuming ≥ 1 serving/week fish, with similar results for immediate and delayed recall. Specifically, for verbal memory; *β* = 0.051 (95% CI: 0.013, 0.088, *p* = 0.008); for immediate recall *β* = 0.046 (0.007, 0.085, *p* = 0.020); for delayed recall *β* = 0.050 (0.013, 0.087, *p* = 0.008), where a positive *β* indicates that the rate of decline is slower for regular fish consumers compared with infrequent consumers [51].

## Discussion

Given the rapid global rise in dementia prevalence and the pressing need for effective prevention strategies, clarifying the role of fish consumption in specific aspects of cognition carries important scientific and public health implications. Accordingly, this systematic review sought to collate and critically evaluate existing evidence on fish consumption and its associations with distinct cognitive domains. In general, evidence suggests that higher fish consumption is more consistently related to processing-speed functions across studies (and, to a lesser extent, verbal/episodic memory). These cognitive functions stand out as the domain with the strongest and most reproducible associations across the cross-sectional and longitudinal evidence. Some studies reported null findings, but heterogeneity of results may also be explained, at least in part, by methodological choices. Studies using quantitative exposure metrics with clear dose-response categories, such as grams per day, servings per week, or intake quantiles, more consistently detected significant associations compared to more generic categorizations. For example, the HUSK study found a dose-dependent association between total seafood intake and cognitive function, with the strongest effects observed at ~ 75 g/day [27]. The CHNS cohort showed that consuming ≥ 1 serving/week was associated with significantly slower cognitive decline compared with lower intake [51]. The SMHS/SWHS cohort found that participants in the highest quintile of fish intake (up to ~ 97 g/day) had significantly lower risk of memory decline and impaired decision-making [50]. The pooled 3C/NHS/WHS/MAP cohorts demonstrated that higher servings per week were associated with slower cognitive decline and memory decline, particularly at ≥ 4 servings/week [43]. In contrast, studies using continuous exposure variables without defined intake categories, dietary scores, or simplified classifications often reported null findings.

The UKWCS study found no significant association when total fish intake was analyzed as a continuous variable [42]. The NUDAD [30] and DELCODE [32] cohorts also found no significant associations using broad fish intake categories or dietary scores. Similarly, some studies using composite dietary indices or limited intake categorization failed to detect associations. This may reflect attenuation due to exposure misclassification or insufficient contrast between intake groups while dose-sensitive categorization improved the ability to detect associations, likely because it captures gradients of exposure and allows threshold effects to emerge. Interestingly, several studies found associations only when analyzing specific fish types rather than total fish intake. The WHS study found that tuna and dark-meat fish intake (≥ 1 serving/week) was associated with significantly better verbal memory, whereas total seafood intake was not associated with cognition [45]. The CARDIA study found significant associations specifically for non-fried seafood consumption, but not for all cognitive outcomes [47]. The HUSK cohort observed the strongest cognitive effects for lean and fatty fish separately, rather than total fish intake alone [27]. This suggests that nutrient-rich fish types (especially fatty fish high in omega-3) may drive the observed associations, and lumping all fish together may dilute detectable effects.

The findings of this review are broadly consistent with earlier studies which have generally reported protective associations between fish or seafood consumption and cognitive outcomes: specifically, a recent meta-analysis of observational studies has shown that higher fish intake is linked to a 20–36% lower risk of dementia and Alzheimer’s disease, with evidence of dose-response relation [19]. By focusing on specific cognitive domains, our review extends our prior work by revealing that fish intake is not only associated with global cognitive outcomes but may differentially influence processing speed functions and verbal/episodic memory, domains highly relevant to the early detection of dementia. The findings of this review reinforce the importance of fish consumption as part of a healthy dietary pattern for the promotion of cognitive health and dementia prevention. Our results are also aligned with most current national dietary recommendations advising regular consumption of fish on a weekly basis for cardiometabolic and overall health benefits, with emerging evidence supporting the extension of these recommendations to brain health [52]. The World Health Organization emphasizes adherence to healthy dietary patterns, including regular fish consumption, as part of a comprehensive strategy to reduce dementia risk [12]. Similarly, the Lancet Commission on Dementia Prevention identifies dietary modification, including greater intake of nutrient-rich foods such as fish, as a critical intervention among the modifiable risk factors for dementia [53]. From a population perspective, modest increases in fish intake could yield substantial benefits, particularly given the projected rise in dementia prevalence worldwide. Our synthesis suggests that adherence to such recommendations may also confer protection against age-related cognitive decline, supporting the inclusion of brain health as an explicit goal of dietary policy.

There is a biologically coherent rationale for the observation that higher fish consumption is more consistently associated with performance in processing speed domains, and to a lesser extent episodic memory, than with other cognitive functions. Processing speed is strongly dependent on the integrity and efficiency of large-scale neural transmission supported by extensively myelinated white matter tracts, including frontoparietal association pathways and subcortical-cortical loops [54–56]. These structures are particularly enriched in docosahexaenoic acid (DHA), the long-chain omega-3 fatty acid most abundant in marine foods [57]. DHA constitutes a major component of myelin and neuronal membranes, where it influences membrane fluidity, axonal conductance, and synaptic signaling [58]. Moreover, DHA exhibits anti-inflammatory and antioxidant properties that help preserve white matter microstructure, which is both highly susceptible to age-related degeneration and tightly coupled to individual differences in processing speed performance [59]. Thus, nutritional exposures that support myelin stability and white matter integrity would be expected to preferentially enhance cognitive functions that rely on rapid neural conduction, providing a mechanistic explanation consistent with the empirical pattern observed across studies. A related mechanism may explain the moderately consistent associations observed for episodic memory. This domain depends principally on hippocampal circuitry, which is also highly enriched in DHA and is vulnerable to oxidative stress, neuroinflammation, and metabolic dysregulation; experimental and human evidence indicates that DHA supports hippocampal synaptic plasticity through effects on long-term potentiation, dendritic spine morphology, and neurotrophin signaling, including the regulation of brain-derived neurotrophic factor (BDNF) [60, 61]. DHA also promotes neurogenesis in the dentate gyrus and modulates membrane excitability in glutamatergic neurons [62, 63]. Collectively, these mechanisms provide a plausible biological basis for the sensitivity of episodic memory processes to dietary omega-3 fatty acid intake, even though inter-study heterogeneity in memory assessments may attenuate the consistency of findings. By contrast, cognitive domains such as executive functions, language, and visuospatial abilities show less consistent associations with fish consumption. These domains rely more heavily on distributed cortical systems in the prefrontal, temporal, and parietal regions, in which DHA is present but which are less dependent on highly myelinated long-range fiber systems [64, 65]. Furthermore, performance in these domains is strongly influenced by educational attainment, lifelong cognitive enrichment, and sociocultural factors, which may overshadow nutritional effects and contribute to weaker or more variable associations [66]. Additionally, the cerebrovascular effects of omega-3 fatty acids offer complementary mechanistic support. Higher adherence to the Mediterranean diet rich in fish is primarily linked to cardiovascular benefits [67–69]. Fish-derived long-chain polyunsaturated fatty acids improve endothelial function, enhance cerebral perfusion, and reduce systemic and neurovascular inflammation [70]. Processing speed measures, which depend on the efficiency of global neural communication and metabolic support, are among the cognitive functions most sensitive to these vascular parameters [71–74]. This vascular sensitivity further reinforces why processing speed, in particular, may show the most robust and reproducible associations with fish consumption across observational and interventional studies. Notably, the “brain–heart axis” framework is supported by experimental and clinical evidence showing that cardiac dysfunction and reduced cerebral perfusion are linked to worse cognition, and that neurotrophin signaling (including BDNF) may participate in bidirectional heart–brain communication [75]. In the “obese + psychosocial stress” mouse model you cite, impaired cardiac performance co-occurred with hippocampal dysfunction and cognitive impairment, and the authors highlighted reduced local BDNF/TrkB-related signaling as a potential mechanistic bridge between cardiac and brain phenotypes [76]. Against this backdrop, fish intake, particularly fatty fish rich in EPA/DHA, could benefit cognition upstream by improving vascular and cardiac determinants of brain health: omega-3s have been shown to improve endothelial function (e.g., flow-mediated dilation) and can lower blood pressure in aggregated evidence, both of which may enhance cerebrovascular reactivity and reduce small-vessel injury that contributes to slower processing speed and executive dysfunction [77]. Beyond hemodynamics, cardioprotective effects (anti-inflammatory, anti-atherogenic, triglyceride-lowering) could reduce systemic vascular inflammation and microvascular dysfunction that compromise neurovascular coupling, mechanisms highly relevant to vascular contributions to cognitive impairment [78]. Finally, BDNF-related signaling is a plausible shared mediator: BDNF is increasingly discussed as a protein linking cardiac rehabilitation, vascular function, and brain plasticity, and there is some evidence from supplementation studies and meta-analyses that omega-3 intake can increase circulating BDNF in certain contexts (though findings are heterogeneous and not uniformly positive) [79]. Taken together, these lines of evidence support the hypothesis that fish consumption may protect cognition partly by stabilizing cardiovascular function and modulating heart–brain signaling (including BDNF/TrkB pathways), thereby preserving cerebral perfusion and reducing vascular brain injury, mechanisms that would complement, rather than replace, direct neuronal effects of fish-derived nutrients.

Emerging evidence suggests that epigenetic mechanisms may represent an additional pathway linking fish consumption to cognitive function, complementing both direct neurobiological and cardiovascular effects [80]. Epigenetic regulation, including DNA methylation, histone modification, and microRNA expression, plays a central role in controlling gene expression relevant to neuronal plasticity, inflammation, and neurodegeneration. Nutrients abundant in fish, particularly omega-3 polyunsaturated fatty acids (PUFAs), have been shown to modulate epigenetic patterns. For example, higher fish and omega-3 intake has been associated with altered DNA methylation of genes involved in lipid metabolism and inflammation, such as ABCA1, suggesting a mechanism through which dietary fish may influence cardiometabolic and vascular pathways relevant to brain health [81]. More broadly, omega-3 fatty acids and other bioactive nutrients can alter DNA methylation and microRNA regulation, thereby influencing inflammatory signaling, oxidative stress responses, and transcriptional control of genes implicated in chronic disease and neurodegeneration [81]. Importantly, dietary factors can also influence epigenetic regulation directly within the central nervous system, as nutrition-dependent changes in DNA methylation and related processes can modify gene expression patterns involved in synaptic plasticity, neurogenesis, and cognitive function [82]. In addition, fish-derived B vitamins (including vitamin B12 and folate) support one-carbon metabolism and methyl donor availability, thereby directly influencing DNA methylation processes that regulate neuronal gene expression and brain aging [83]. These findings support the hypothesis that epigenetic mechanisms may partly mediate the relationship between fish consumption and cognitive resilience by modulating gene expression pathways involved in neurotrophic signaling, vascular function, and neuroinflammation. Given that epigenetic modifications are dynamic and potentially reversible, this represents a particularly promising avenue for future research. Longitudinal and interventional studies incorporating epigenomic profiling (such as genome-wide DNA methylation or circulating microRNA analyses) alongside dietary assessment and cognitive phenotyping could help clarify causal pathways and identify novel biomarkers linking fish intake to brain health. Such integrative approaches may ultimately enable precision nutrition strategies aimed at preserving cognitive function through modulation of the epigenome.

There are several components of fish that may explain the biological effects of the whole food matrix toward brain health and cognitive function. One of the most widely studied mechanisms linking fish intake to cognitive health is its high content of long-chain omega-3 PUFAs, primarily DHA and EPA [57]. DHA is a structural component of neuronal membranes, accounting for up to 40% of PUFAs in the gray matter, where it modulates membrane fluidity, receptor function, and synaptic plasticity [58]. Both DHA and EPA exert anti-inflammatory effects by downregulating pro-inflammatory cytokines and generating specialized pro-resolving mediators, thereby protecting neurons from chronic neuroinflammation implicated in Alzheimer’s disease [59]. In addition, omega-3 PUFAs enhance neurogenesis and synaptic connectivity, partly through upregulation of BDNF and modulation of neurotransmitter systems, including dopaminergic and serotonergic pathways, which are critical for memory and executive function [57, 60, 61]. Animal models have shown that DHA supplementation improves long-term potentiation, a physiological correlate of learning and memory [62], while human studies associate higher circulating omega-3 levels with larger hippocampal volumes and slower cognitive decline [84, 85].

Other fish components, albeit not targeting specific cognitive domains, may collectively support the hypothesis that fish consumption is related to better cognitive function. One important bioactive component of fish relevant to brain health is vitamin D, which is abundant in fatty fish such as salmon, mackerel, and sardines [86]. Vitamin D receptors and the enzyme 1α-hydroxylase are widely expressed in the brain, particularly in regions critical for cognition such as the hippocampus and prefrontal cortex [87, 88] suggesting a direct neuromodulatory role [89, 90]. Experimental studies indicate that vitamin D contributes to neuroprotection by regulating calcium homeostasis, reducing oxidative stress, and modulating the expression of neurotrophic factors, including nerve growth factor (NGF) and glial cell line-derived neurotrophic factor (GDNF) [91]. In addition, vitamin D downregulates the production of pro-inflammatory cytokines and promotes clearance of amyloid-β peptides, mechanisms directly implicated in Alzheimer’s disease pathogenesis [92]. Epidemiological studies have linked low serum 25(OH)D concentrations with poorer increased dementia and Alzheimer’s disease risk [93], although causality remains debated due to potential confounding by comorbidities and lifestyle. Selenium, another micronutrient richly supplied by fish and seafood [94, 95], has been increasingly recognized for its potential neuroprotective properties. As an essential trace element, selenium is incorporated into selenoproteins such as glutathione peroxidases and thioredoxin reductases, which play central roles in maintaining redox balance and protecting neurons from oxidative damage [96]. Beyond antioxidant defense, selenium influences thyroid hormone metabolism [97] and immune regulation [98], both of which are relevant to brain development and cognitive function [99, 100]. Observational studies have shown that low plasma selenium concentrations are associated with poorer memory performance, accelerated cognitive decline, and higher risk of dementia in older adults [101–106]. Moreover, animal experiments suggest that selenium supplementation enhances hippocampal synaptic plasticity and may counteract amyloid-β neurotoxicity [107–109]. While human intervention trials remain limited, the cumulative evidence supports a role for adequate selenium intake, potentially through fish consumption, in sustaining cognitive resilience during aging. Iodine, naturally abundant in many marine fish and seafood [110], is a crucial micronutrient for optimal brain function due to its role in thyroid hormone synthesis [111]. Thyroid hormones regulate neuronal differentiation, myelination, and synaptic plasticity, processes that remain relevant not only in early development but also for maintaining cognitive function across the lifespan [112, 113]. Severe iodine deficiency during pregnancy and childhood is a well-established cause of irreversible neurodevelopmental impairment [114], but emerging evidence also suggests that even mild to moderate deficiency in adults may adversely affect cognitive performance [38]. Furthermore, inadequate iodine status may exacerbate age-related cognitive decline indirectly by altering thyroid function, which has been linked to dementia risk [115, 116]. Fish is also a valuable dietary source of B vitamins, particularly vitamin B12 and B6, and also provides small amounts of folate [117], which are essential for one-carbon metabolism and maintenance of neurological function [118]. Both vitamins are critical cofactors in homocysteine metabolism, and deficiencies can lead to elevated homocysteine levels [119], a recognized risk factor for vascular disease, brain atrophy, and cognitive decline [120, 121]. Vitamin B12 also contributes to myelin synthesis and neurotransmitter production [122, 123], processes directly implicated in memory and executive function [124, 125]. Epidemiological studies have shown that low serum concentrations of B12 and folate, among others, are associated with poorer cognitive performance, accelerated decline, and higher dementia risk [126]. Moreover, randomized controlled trials indicate that B vitamin supplementation can slow the rate of brain atrophy and cognitive deterioration in older adults with mild cognitive impairment, particularly in those with elevated homocysteine levels [127]. While the effects may be modest and context-dependent, these findings support the hypothesis that fish-derived B vitamins may act synergistically with other nutrients such as omega-3 fatty acids to promote brain health and reduce the risk of cognitive decline.

The current body of evidence provides consistent support for a beneficial association between fish consumption and cognitive health; however, some methodological limitations must be acknowledged. First, most of the included studies are observational in design, which limits causal inference due to the potential for residual confounding. Socioeconomic status, education, overall diet quality, and health-conscious behaviors often correlate with higher fish intake and may partially account for observed associations. This is also suggested in some reports reporting positive associations in unadjusted or minimally adjusted models that are attenuated or rendered non-significant after full covariate adjustment: this pattern suggests sensitivity of findings to confounder control and raises concerns regarding the robustness of some reported associations that need to be further clarified. Moreover, cross-sectional and shorter follow-up studies may further suffer from reverse causation, as individuals with better cognitive status may be more likely to maintain healthier dietary habits (healthy-user bias), including higher fish consumption, rather than fish intake directly influencing cognitive outcomes. This concern is particularly relevant in older populations, where early, subclinical cognitive decline may lead to changes in dietary behaviors, food preparation ability, or appetite, potentially biasing observed associations. In addition, short follow-up periods may be insufficient to capture the long latency of neurodegenerative processes, which develop over decades. Consequently, studies with limited follow-up may underestimate the long-term protective effects of sustained fish consumption. The scarcity of long-term prospective studies also limits the ability to identify critical exposure windows, such as midlife versus late-life intake, or to evaluate cumulative exposure models that may be more relevant for neuroprotection. Future longitudinal studies with repeated dietary assessments across the life course and extended follow-up durations are needed to better characterize temporal relationships, reduce reverse causation bias, and clarify whether sustained fish consumption contributes to cognitive resilience over time. Second, dietary exposures were frequently assessed through FFQ or self-reported intake, while others used 24-h dietary recalls, short-term food records, or brief questionnaires with limited dietary detail, which are prone to recall bias and misclassification. FFQs, although useful for capturing habitual intake over extended periods, are subject to recall bias and systematic measurement error, particularly in older populations where cognitive impairment itself may affect reporting accuracy. Moreover, FFQs vary considerably in length and specificity; for example, some studies used detailed instruments with over 170 food items and differentiation between fish types (i.e., oily vs. lean fish), whereas others relied on shorter questionnaires or single-item measures, which may inadequately capture variability in intake. Notably, studies employing repeated or more detailed dietary assessments (such as multi-day dietary records or repeated 24-h recalls) were more likely to detect significant associations between fish consumption and cognitive outcomes, whereas studies using simplified questionnaires or single-time assessments more frequently reported null findings. Importantly, measurement error in dietary assessment is likely to be largely nondifferential with respect to future cognitive outcomes in prospective studies, which would tend to bias associations toward the null and potentially obscure true protective effects of fish consumption. Furthermore, differences in how fish exposure was defined (including total fish intake, fatty versus lean fish, or seafood consumption frequency) may also contribute to heterogeneity in findings, as these categories differ substantially in nutrient composition. Collectively, these methodological limitations underscore the importance of using validated, detailed, and repeated dietary assessment tools, as well as objective biomarkers of fish intake where feasible, to improve exposure characterization and strengthen causal inference in future research. Third, the cognitive outcomes themselves were heterogeneous, with variability in test batteries, diagnostic criteria, and domain classification, limiting comparability across studies. Different cognitive tests vary in their sensitivity, specificity, and susceptibility to ceiling or floor effects, which may influence their ability to detect subtle cognitive differences associated with dietary exposures. As a result, studies using less sensitive or domain-limited assessments may underestimate true associations or contribute to null findings. Furthermore, inconsistencies in how cognitive domains are defined and operationalized may lead to misclassification, obscuring potential domain-specific effects of fish consumption. Greater harmonization of cognitive assessment protocols, including the use of standardized and domain-sensitive neuropsychological batteries and validated composite scores, would improve comparability across studies and enhance the ability to detect meaningful associations. Fourth, intervention trials remain scarce, and many have tested omega-3 supplementation rather than whole-fish consumption, which may underestimate the synergistic effects of the multiple nutrients present in seafood. Finally, most studies did not differentiate between Alzheimer-type and vascular cognitive impairment, limiting etiological specificity. In fact, fish consumption is hypothesized to exert beneficial effects on cardiovascular and cerebrovascular health, which may mediate, at least in part, the putative effects on cognitive function through vascular mechanisms rather than direct effects on neurodegenerative processes. Ultimately, this limitation does not undermine the potential clinical outcome toward a better cognitive function associated with fish consumption, whether confirmed. Despite these limitations, the evidence base has several strengths. The included studies span diverse populations across continents, increasing generalizability. Many are large-scale, prospective cohorts with long follow-up periods, allowing temporal relationships to be assessed. Moreover, consistent findings across different study designs and settings, along with biologically plausible mechanisms, lend credibility to the observed associations. Together, these factors provide a strong rationale for further clinical and mechanistic studies focusing specifically on fish intake and its role in preserving cognitive function.

Based on these premises, future research should consider specific methodological aspects in order to overcome the aforementioned limitations and strengthen the evidence on the putative beneficial association between fish intake and cognitive outcomes. First, high-quality randomized controlled trials directly testing fish consumption, rather than omega-3 supplementation alone, are highly warranted. Such trials are necessary to determine whether the benefits observed in observational cohorts are causal and to quantify optimal intake levels. Second, future studies should differentiate between types of fish (e.g., oily vs. lean, freshwater vs. marine) and preparation methods, as nutrient profiles and contaminant burdens vary widely. Third, standardized approaches to measuring both dietary exposure and cognitive outcomes are urgently needed. Harmonized food-frequency tools, biomarkers of nutrient intake (e.g., plasma omega-3, selenium, vitamin B12), and domain-specific cognitive batteries would improve comparability across studies. Another priority is to explore subgroup differences: whether benefits are more pronounced in individuals at higher genetic risk (e.g., APOE ε4 carriers), with nutritional deficiencies, or in populations from low- and middle-income countries where both fish consumption patterns and dementia burdens differ. Future studies should incorporate more precise phenotyping of cognitive impairment, including clinical adjudication of dementia subtypes, neuroimaging markers of cerebrovascular disease (e.g., white matter hyperintensities, infarcts, microbleeds), and biomarkers of Alzheimer’s pathology, such as amyloid-β and tau in cerebrospinal fluid or plasma. Longitudinal studies integrating dietary assessment with multimodal biomarkers and neuroimaging would allow researchers to disentangle vascular and neurodegenerative pathways and clarify the specific mechanisms through which fish consumption may preserve cognitive function. Moreover, assessing long-term fish consumption might help identify the role of dose and time cumulative exposure in order to draft proper age-specific dietary recommendations. In addition, equity considerations are critical: access to affordable, high-quality fish varies globally, and promoting fish consumption may be challenging in low-resource settings or regions where plant-based diets dominate. Finally, translational research is needed to integrate dietary recommendations into multidomain prevention programs and to evaluate real-world feasibility, sustainability, and equity of increasing fish consumption globally. Collectively, these findings highlight the potential of dietary strategies centered on fish and seafood to form a low-cost, scalable, and culturally adaptable component of dementia prevention initiatives, complementing other lifestyle interventions such as physical activity, smoking cessation, and vascular risk management. Addressing these gaps will strengthen the evidence base and help translate nutritional epidemiology into actionable strategies for preserving cognitive health across aging populations.

In conclusion, this systematic review supports, at least in part, the evidence that higher fish consumption is associated with better cognitive performance across several domains, which may contribute to reduced risk of cognitive decline and dementia. Overall, convergent neurobiological, vascular, and cognitive-aging scientific literature supports the interpretation that fish consumption exerts its most detectable and consistent effects on processing speed functions, followed by episodic memory, with more limited and heterogeneous effects observed in other cognitive domains. The beneficial effects are biologically plausible, supported by multiple mechanisms involving omega-3 fatty acids, vitamin D, selenium, iodine, and B vitamins, which collectively promote neuronal integrity, synaptic function, and neuroprotection. However, randomized controlled trials directly testing fish consumption (as opposed to omega-3 supplementation) remain scarce: with most studies to date being observational in nature and limited by methodological heterogeneity, the overall strength of the evidence remains moderate due to inconsistency across findings. Nonetheless, by situating our results within the context of previous reviews and established guidelines, this study strengthens the case for fish as a cornerstone of dietary strategies to preserve cognitive function and highlights the value of incorporating cognitive outcomes into future iterations of nutritional policy. Future high-quality studies are still needed to confirm causality, by providing proofs of effects in the elderly population via intervention studies, as well as clarifying dose–response relationships to optimize public health recommendations. Based on the domain-specific findings of this review, episodic memory, executive function, and processing speed emerge as the most relevant cognitive domains associated with fish consumption and should be prioritized as primary endpoints in future randomized trials. Episodic memory, in particular, appears to be the most sensitive domain, consistent with its central role in early neurodegenerative processes and its responsiveness to nutritional exposures. Global cognitive composite scores may also serve as valuable complementary endpoints due to their robustness and clinical relevance. Prioritizing these domain-sensitive and biologically meaningful outcomes will enhance the ability of future trials to detect intervention effects and clarify the role of fish consumption in preserving cognitive health.

## Supplementary Information

Below is the link to the electronic supplementary material.

ESM1 (DOCX 3.09 MB)

## Data Availability

Not applicable.
