# Supplementary material for: Fish consumption and cognitive function in aging: a systematic review of observational studies
Source: GeroScience. 2026 Mar 15;48(3):4923–56. doi: 10.1007/s11357-026-02188-w (PMC13356019; doi:10.1007/s11357-026-02188-w)

Supplementary Table 1. The Meta-analysis of Observational Studies in Epidemiology (MOOSE) guidelines.

| **Section/topic** | **#** | **Checklist item** | **Reported on page #** |
| --- | --- | --- | --- |
| **TITLE** | | |  |
| Title | 1 | Identify the report as a systematic review, meta-analysis, or both. | 1 |
| **ABSTRACT** | | |  |
| Structured summary | 2 | Provide a structured summary including, as applicable: background; objectives; data sources; study eligibility criteria, participants, and interventions; study appraisal and synthesis methods; results; limitations; conclusions and implications of key findings; systematic review registration number. | 2 |
| **INTRODUCTION** | | |  |
| Rationale | 3 | Describe the rationale for the review in the context of what is already known. | 3-5 |
| Objectives | 4 | Provide an explicit statement of questions being addressed with reference to participants, interventions, comparisons, outcomes, and study design (PICOS). | 5 |
| **METHODS** | | |  |
| Protocol and registration | 5 | Indicate if a review protocol exists, if and where it can be accessed (e.g., Web address), and, if available, provide registration information including registration number. | 5 |
| Eligibility criteria | 6 | Specify study characteristics (e.g., PICOS, length of follow-up) and report characteristics (e.g., years considered, language, publication status) used as criteria for eligibility, giving rationale. | 5, Table S3 |
| Information sources | 7 | Describe all information sources (e.g., databases with dates of coverage, contact with study authors to identify additional studies) in the search and date last searched. | 5 |
| Search | 8 | Present full electronic search strategy for at least one database, including any limits used, such that it could be repeated. | 5, Table S2 |
| Study selection | 9 | State the process for selecting studies (i.e., screening, eligibility, included in systematic review, and, if applicable, included in the meta-analysis). | 5, Fig.S1 |
| Data collection process | 10 | Describe method of data extraction from reports (e.g., piloted forms, independently, in duplicate) and any processes for obtaining and confirming data from investigators. | 6 |
| Data items | 11 | List and define all variables for which data were sought (e.g., PICOS, funding sources) and any assumptions and simplifications made. | 6 |
| Risk of bias in individual studies | 12 | Describe methods used for assessing risk of bias of individual studies (including specification of whether this was done at the study or outcome level), and how this information is to be used in any data synthesis. | 6 |
| Summary measures | 13 | State the principal summary measures (e.g., risk ratio, difference in means). | NA |
| Synthesis of results | 14 | Describe the methods of handling data and combining results of studies, if done, including measures of consistency (e.g., I^2^) for each meta-analysis. | NA |
| Risk of bias across studies | 15 | Specify any assessment of risk of bias that may affect the cumulative evidence (e.g., publication bias, selective reporting within studies). | NA |
| Additional analyses | 16 | Describe methods of additional analyses (e.g., sensitivity or subgroup analyses, meta-regression), if done, indicating which were pre-specified. | NA |
| **RESULTS** | | |  |
| Study selection | 17 | Give numbers of studies screened, assessed for eligibility, and included in the review, with reasons for exclusions at each stage, ideally with a flow diagram. | 6, Fig. S1 |
| Study characteristics | 18 | For each study, present characteristics for which data were extracted (e.g., study size, PICOS, follow-up period) and provide the citations. | 6-14, Table 1 |
| Risk of bias within studies | 19 | Present data on risk of bias of each study and, if available, any outcome level assessment (see item 12). | Table S5, Table S6 |
| Results of individual studies | 20 | For all outcomes considered (benefits or harms), present, for each study: (a) simple summary data for each intervention group (b) effect estimates and confidence intervals, ideally with a forest plot. | 6-14, Table 1 |
| Synthesis of results | 21 | Present results of each meta-analysis done, including confidence intervals and measures of consistency. | NA |
| Risk of bias across studies | 22 | Present results of any assessment of risk of bias across studies (see Item 15). | NA |
| Additional analysis | 23 | Give results of additional analyses, if done (e.g., sensitivity or subgroup analyses, meta-regression [see Item 16]). | NA |
| **DISCUSSION** | | |  |
| Summary of evidence | 24 | Summarize the main findings including the strength of evidence for each main outcome; consider their relevance to key groups (e.g., healthcare providers, users, and policy makers). | 14-19 |
| Limitations | 25 | Discuss limitations at study and outcome level (e.g., risk of bias), and at review-level (e.g., incomplete retrieval of identified research, reporting bias). | 19,20 |
| Conclusions | 26 | Provide a general interpretation of the results in the context of other evidence, and implications for future research. | 20,21 |
| **FUNDING** | | |  |
| Funding | 27 | Describe sources of funding for the systematic review and other support (e.g., supply of data); role of funders for the systematic review. | 21 |

Table S2. Systematic search strategy

| Pubmed | |
| --- | --- |
|  | ("fishes"[MeSH Terms] OR "fishes"[All Fields] OR "fish"[All Fields] OR "seafood"[MeSH Terms] OR "seafood"[All Fields] OR "seafoods"[All Fields] OR "shellfish"[MeSH Terms] OR "shellfish"[All Fields] OR "shellfishes"[All Fields]) AND (“cognitive dysfunction"[MeSH Terms] OR "cognition"[MeSH Terms] OR "cognition"[All Fields] OR "cognitions"[All Fields] OR "cognitive"[All Fields] OR "cognitively"[All Fields] OR "cognitives"[All Fields] OR "memories"[All Fields] OR "memory"[MeSH Terms] OR "memory"[All Fields] OR "memory s"[All Fields] OR "dementia"[MeSH Terms] OR "dementia"[All Fields] OR "dementias"[All Fields] OR "dementia s"[All Fields] OR "alzheime s"[All Fields] OR "alzheimer disease"[MeSH Terms] OR ("alzheimer"[All Fields] AND "disease"[All Fields]) OR "alzheimer disease"[All Fields] OR "alzheimer"[All Fields] OR "alzheimers"[All Fields] OR "alzheimer s"[All Fields] OR "alzheimers s"[All Fields] ​​OR "neurodegenerative"[All Fields] OR "neurodegeneration"[All Fields] OR "Neurodegenerative Diseases"[MeSH Terms] OR "intellectual impairment"[All Fields] OR "intellectual dysfunction"[All Fields]) AND ("prospective"[All Fields] OR "prospectively"[All Fields] OR "longitudinal"[All Fields] OR "longitudinally"[All Fields] OR "cohort"[All Fields] OR "cohort s"[All Fields] OR "cohorte"[All Fields] OR "cohorts"[All Fields] OR "observational"[All Fields] OR "follow-up"[All Fields] OR "nested"[All Fields] OR "case-control"[All Fields] OR "cross-sectional"[All Fields] OR "population-based"[All Fields] OR "Cohort Studies"[MeSH Terms] OR "Longitudinal Studies"[MeSH Terms] OR "Prospective Studies"[MeSH Terms] OR "Cross-Sectional Studies"[MeSH Terms] OR "Case-Control Studies"[MeSH Terms] OR "Epidemiologic Studies"[MeSH Terms] OR "Longitudinal Studies"[MeSH Terms] OR "Observational Study"[Publication Type]) |
| Scopus | |
|  | TITLE-ABS-KEY ( ( fish OR seafood OR shellfish ) AND ( cognitive OR memory OR dementia OR alzheimer's OR alzheimer ) AND ( prospective OR prospectively OR longitudinal OR cohort OR cohorts OR observational OR follow-up OR nested OR case-control OR cross-sectional ) ) |

Table S3. Population, Intervention, Comparison, Outcomes and Study (PICOS) criteria

| **Parameter** | **Description** |
| --- | --- |
| (P) Population | Older adults |
| (I) Intervention/Exposure | Habitual fish, seafood or shellfish consumption |
| (C) Comparison | Different categories of habitual fish, seafood or shellfish consumption |
| (O) Outcome | Cognitive function |
| (S) Study design | Observational studies with a comparison group (cohort studies, cross-sectional studies, case-control studies) |

Table S4. List of tests used to assess various cognitive domains

| **Domain** | **Test (Acronym)** |
| --- | --- |
| **Memory** | Kendrick Object Learning Test (KOLT) |
|  | Verbal Learning Test (VLT) |
|  | Story Recall Test (SRT) |
|  | Selective Reminding Test (SRT — variant) |
|  | Rey Auditory Verbal Learning Test (RAVLT) |
|  | Visual Association Test (VAT) |
|  | Word Memory |
|  | Face Memory |
|  | Logical Memory |
|  | East Boston Memory Test (EBMT) |
|  | East Boston Story |
|  | Word List Memory (WLM) |
|  | Word List Recall (WLR) |
|  | Word List Recognition (WLRc) |
|  | CERAD Word List Learning |
|  | Visual Reproduction Test (VRT) |
|  | 5-Word Test |
|  | RI-48 Cued Recall Test (RI-48) |
| **Executive Function** | Controlled Oral Word Association Test (COWAT) |
|  | Concept Shifting Test (CST) |
|  | Stroop Color–Word Test (SCWT) |
|  | Stroop Neuropsychological Screening Test |
|  | Colour Stroop Test (Colour ST) |
|  | Spatial Stroop Test (Spatial ST) |
|  | Simon Task |
|  | Flanker Task (FT) |
|  | Verbal Fluency Test (VFT) |
|  | Category Fluency Test (CFT) |
|  | Letter Fluency Test (LFT) |
|  | Everyday Problems Test (EPT) |
|  | Odd-Man-Out Test (OMO) |
|  | Frontal Assessment Battery (FAB) |
|  | Retrieval Fluency |
| **Processing Speed** | Trail Making Test Part A (TMT-A) |
|  | Digit Symbol Test (DST / DSST / m-DST) |
|  | Letter–Digit Substitution Test (LDST) |
|  | Symbol Digit Modalities Test (SDMT) |
|  | Inspection Time |
|  | Pattern Comparison Test |
|  | Number Comparison Test |
| **Attention / Working Memory** | Digit Span Forward (DS-F) |
|  | Digit Span Backward (DS-B) |
|  | Digit Ordering Test |
|  | Continuous Performance Test (CPT) |
|  | Sternberg Memory Scanning (SMS) |
|  | Counting Span |
|  | Operation Span |
| **Language** | Boston Naming Test (BNT) |
|  | Boston Naming Test – short version (BNT-short) |
|  | Vocabulary (WAIS-R) |
|  | 15-item Reading Test |
|  | Word Endings (KFRCT subtest) |
| **Visuospatial** | Block Design (BD / m-BD) |
|  | Visual Object and Space Perception battery (VOSP) |
|  | Constructional Praxis (CP) |
|  | Judgment of Line Orientation (JLO) |
|  | Location Memory |
|  | Object Assembly (WAIS) |
|  | Picture Completion (WAIS) |
|  | Picture Arrangement (WAIS) |
| **Fluid Reasoning** | Standard Progressive Matrices (SPM) |
|  | Standard Progressive Matrices Plus (SPM+) |
|  | Matrix Reasoning (WAIS-IIIUK) |
|  | Letter Sets (KFRCT subtest) |
|  | Number Comparison (KFRCT subtest) |
| **Global Cognition** | Mini-Mental State Examination (MMSE) |
|  | Modified MMSE (m-MMSE) |
|  | Montreal Cognitive Assessment (MoCA) |
|  | Telephone Interview for Cognitive Status (TICS) |
|  | Telephone Interview for Cognitive Status – modified (TICS-m) |
| **Psychomotor / Reaction Time** | Simple Reaction Time (SRT) |
|  | Choice Reaction Time (CRT) |
|  | Movement Time Tests – Simple, Up, Diagonal (MT) |
| **Multi-Domain Batteries** | Wechsler Adult Intelligence Scale (WAIS / WAIS-R / WAIS-IIIUK) |
|  | Consortium to Establish a Registry for Alzheimer’s Disease (CERAD battery) |
|  | Kit of Factor-Referenced Cognitive Tests (KFRCT) |
|  | EPOCH Cognitive Battery (comprehensive multi-domain) |

Table S5. Assessment of study quality according to the Newcastle-Ottawa Quality Assessment Scale for cross-sectional studies

|  | SELECTION | | | COMPARABILITY | OUTCOME | | TOTAL SCORE |
| --- | --- | --- | --- | --- | --- | --- | --- |
| Name, year | Representativeness of the exposed cohort | Non-respondents | Ascertainment of exposure |  | Assessment of outcome | Statistical test |  |
| Kalmijn, 2004 | ★ | ★ | ★ | ★★ | ★ | ★ | 7 |
| Nurk, 2007 | ★ | ★ | ★ | ★★ | ★ | ★ | 7 |
| Dangour, 2009 |  |  |  | ★★ | ★ | ★ | 4 |
| Danthiir, 2014 |  |  | ★ | ★★ | ★ | ★ | 5 |
| Brouwer-Brolsma, 2018 | ★ |  | ★ | ★★ | ★ | ★ | 6 |
| Fieldhouse, 2020 |  |  | ★ | ★★ | ★ | ★ | 5 |
| Huang b, 2021 | ★ |  | ★ | ★★ | ★ | ★ | 6 |
| Rauchmann, 2023 |  |  | ★ | ★★ | ★ | ★ | 5 |

Table S6. Assessment of study quality according to the Newcastle-Ottawa Quality Assessment Scale for prospective studies

|  | SELECTION | | | | COMPARABILITY | OUTCOME | | | TOTAL SCORE |
| --- | --- | --- | --- | --- | --- | --- | --- | --- | --- |
| Name, year | Representativeness of the exposed cohort | Selection of the non-exposed cohort | Ascertainment of exposure | Demonstration that outcome of interest was not present at start of study |  | Assessment of outcome | Was follow-up long enough for outcomes to occur | Adequacy of follow up of cohorts |  |
| Van de Rest, 2009 |  | ★ |  | ★ | ★★ | ★ | ★ |  | 6 |
| Kesse-Guyot, 2011 |  | ★ | ★ |  | ★★ | ★ |  |  | 7 |
| Kim, 2013 |  | ★ |  | ★ | ★★ | ★ | ★ | ★ | 7 |
| Samieri, 2013 |  | ★ | ★ | ★ | ★★ | ★ | ★ |  | 7 |
| Kesse-Guyot, 2014 |  | ★ |  |  | ★★ | ★ |  |  | 4 |
| Qin, 2014 | ★ | ★ | ★ |  | ★★ | ★ | ★ |  | 7 |
| Crichton, 2015 | ★ | ★ | ★ |  | ★★ |  | ★ |  | 6 |
| Van De Rest, 2016 |  | ★ | ★ | ★ | ★★ | ★ | ★ | ★ | 8 |
| Hernández, 2017 |  | ★ |  | ★ | ★ | ★ | ★ |  | 5 |
| Fischer, 2018 | ★ | ★ | ★ |  | ★★ | ★ | ★ |  | 7 |
| Nooyens, 2018 | ★ | ★ |  |  | ★★ | ★ | ★ |  | 6 |
| Samieri, 2018 | . | ★ | ★ | ★ | ★★ | ★ | ★ | ★ | 8 |
| Zhu, 2018 | ★ | ★ | ★ |  | ★★ |  |  |  | 5 |
| Mao, 2019 | ★ | ★ | ★ |  | ★★ | ★ | ★ |  | 7 |
| Zhang, 2021 |  | ★ |  |  | ★★ | ★ | ★ |  | 5 |
| Ylilauri, 2022 | ★ | ★ | ★ | ★ | ★★ | ★ | ★ |  | 8 |
| Sasaki, 2024 | ★ | ★ | ★ | ★ | ★★ | ★ | ★ |  | 8 |

Figure S1. Flow chart of the study selection process


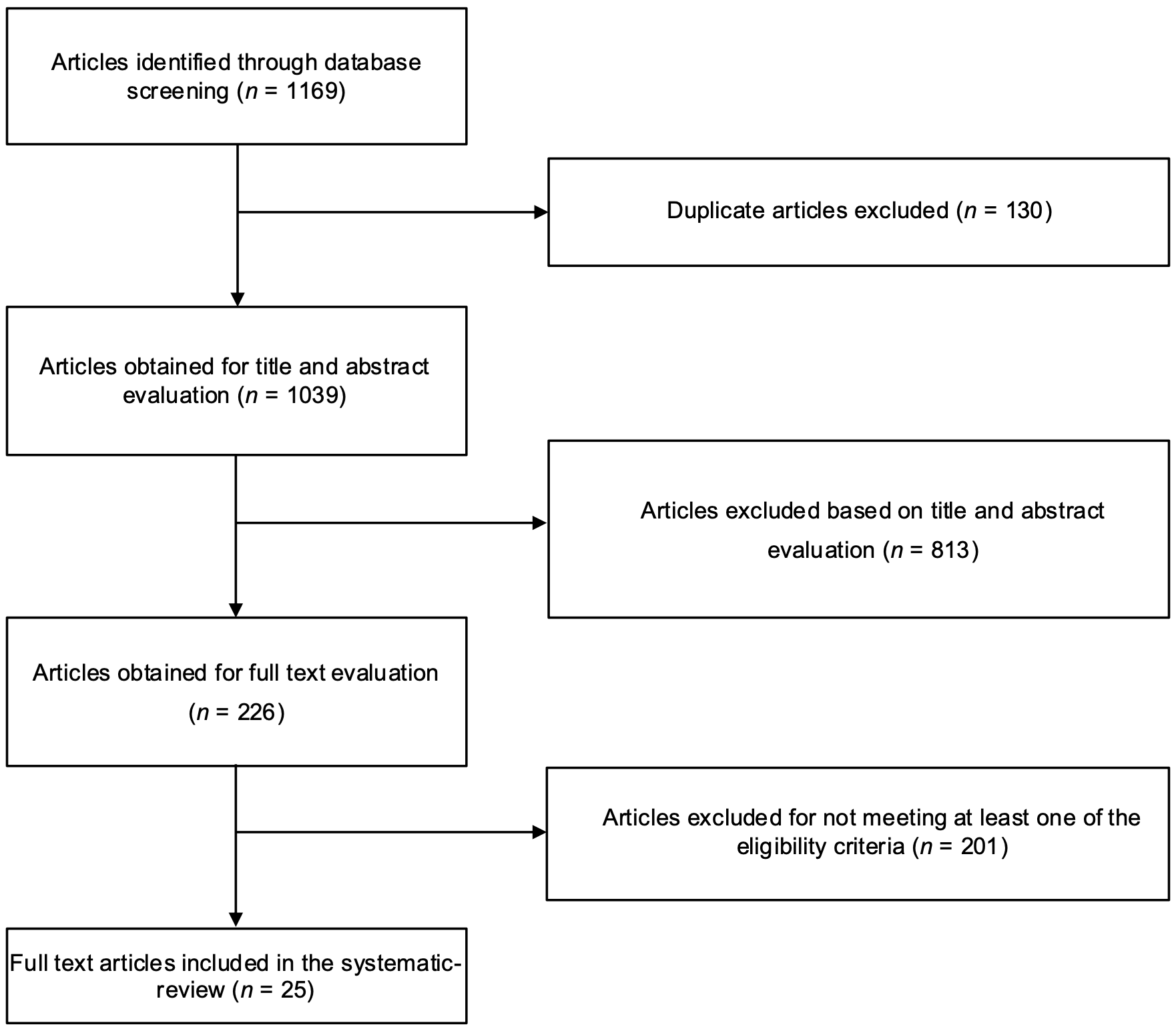

Supplement: Supplementary file 1 — (DOCX 3.09 MB) [file 11357_2026_2188_MOESM1_ESM.docx]
